# The effects of a highly bioavailable curcumin Phytosome^TM^ preparation on the retinal architecture and glial reactivity in the GFAP-IL6 mice

**DOI:** 10.3389/fopht.2023.1205542

**Published:** 2023-10-06

**Authors:** Víctor Pérez-Fernández, Akshaya Lakshmi Thananjeyan, Faheem Ullah, Gerald Münch, Morven Cameron, Erika Gyengesi

**Affiliations:** ^1^ Department of Anatomy and Cell Biology, Western Sydney University, Campbelltown, NSW, Australia; ^2^ Department of Pharmacology, Western Sydney University, Campbelltown, NSW, Australia; ^3^ Neurosurgery, Robert Wood Johnson Medical School, Rutgers University, Piscataway, NJ, United States

**Keywords:** anti-inflammatory, glial activation, retinal morphology, curcumin Phytosome^TM^, retinal microglia

## Abstract

Uncontrolled, chronic inflammation in the retina can disturb retinal structure and function leading to impaired visual function. For the first time, in a mouse model of chronic neuroinflammation (GFAP-IL6), we investigated the impact of chronic glial activation on the retinal microglia population and structure. In addition, we tested a curcumin Phytosome^TM^ preparation with enhanced bioavailability to investigate the effects of a cytokine-suppressing anti-inflammatory drug on retinal architecture. Curcumin Phytosome^TM^ was fed to 3-month old GFAP-IL6 mice for 4 weeks and compared to their untreated GFAP-IL6 counterparts as well as wild type mice on control diet. Microglial numbers and morphology together with neuronal numbers were characterized using immunohistochemistry and cell reconstruction in the retina, using retinal wholemount and slices. GFAP-IL6 mice showed a significant increase in Iba1-labelled mononuclear phagocytes, including microglia, and displayed altered glial morphology. This resulted in a reduction in cone density and a thinning of the retinal layers compared to wild type mice. Curcumin Phytosome^TM^ treatment contributed to decreased microglial density, significantly decreasing both soma and cell size compared to control diet, as well as preventing the thinning of the retinal layers. This study is the first to characterize the impact of chronic retinal inflammation in the GFAP-IL6 mouse and the therapeutic benefit of enhanced bioavailable curcumin Phytosome^TM^ to significantly reduce microglia density and prevent neuronal loss. These data suggest that curcumin could be used as a complementary therapy alongside traditional treatments to reduce associated retinal inflammation in a variety of retinal diseases.

## 1 Introduction

In the retina, chronic neuroinflammation is associated with retinal degeneration and is commonly seen in retinal degenerative diseases such as macular degeneration, diabetic retinopathy, retinal vein occlusion, retinitis pigmentosa and glaucoma (1–3). Although the origin of these retinal diseases is multifactorial, increasing evidence points to microglia as a key player in the initiation, propagation, and aggravation of these disorders due to excessive release of pro-inflammatory mediators that trigger high oxidative stress, and ultimately result in neuronal loss and vision impairment (4–6). The retina, an extension of the brain, is often used as an accessible proxy to study central nervous system (CNS) disorders. Examining the alterations of this tissue, and its various cell types, in response to chronic inflammation could provide insights into the mechanisms of inflammation in the rest of the brain. Furthermore, the retina is a highly organized tissue with well-defined layers, making it easier to visualize and analyze changes in cellular morphology and function.

Inflammation of neural tissue is correlated with dysregulation of microglia which is increased in density in animal models of retinitis pigmentosa (7–9), glaucoma (10) diabetic retinopathy (11) and retinal ischemia (12). In addition, microglia have been shown to be abnormally distributed across retinal layers in different retinal diseases with a certain preference for the retinal ganglion cell layer (1, 10, 13, 14). Microglia can present different phenotypes depending on their activation state; while the resting state presents ramified dendritic arbours that continuously scan the environment, reactive microglia exhibit de-ramification, amoeboid shape and enlarged cell bodies (15). In the retina, activation of microglia can adopt two distinct phenotypes: classically activated (M1), and “alternatively” activated (M2) (11, 16–18). While M1 activation is accompanied by the release of pro-inflammatory cytokines such as IL-1, IL-6 and TNF-α (19), M2 activation preferentially increases phagocytosis and triggers anti-inflammatory cytokines such as IL-4, IL-10 and IFN-α (20). Suppression of this microglial activation has been shown to be neuroprotective in some mouse models of retinal degeneration and thus represents an important therapeutic target (21–25). The M1/M2 dichotomy, while commonly used and somewhat useful, is an oversimplified framework that portrays microglial activation in two extreme states. In reality, the functional phenotypes of microglia in the CNS *in vivo* encompass a diverse spectrum with overlapping characteristics (26).

Our lab has extensively characterized the GFAP-IL6 mouse, a mouse model of chronic neuroinflammation, where interleukin-6 (IL6) is constitutively expressed under a glial fibrillary acidic protein (GFAP) promotor in astroglia (27–31). Chronic overexpression of cerebral and cerebellar IL-6 in this mouse strain triggers several neuropathologies (32), and GFAP-IL6 mice exhibit significant abnormalities in glial activation, behavior, and neurophysiology (33). However, this mouse model has never been used to study the retina, despite IL-6 being associated with retinal inflammation (34), diabetic retinopathy (11), and glaucoma (35, 36).

Anti-inflammatory compounds such as curcumin not only attenuate the reactive state of microglia but also return them to their anti-inflammatory phenotype (37–39). Furthermore, curcumin has been shown to ameliorate retinal degeneration associated with inflammation in different disease backgrounds (40–43). However, one of the major drawbacks of curcumin is its poor absorption, biodistribution, metabolism, and bioavailability. Several formulations have tried to overcome some of these downsides (reviewed in (44)), among these, phytosome-encapsulated curcumin preparation enhanced both the absorption and the levels of curcumin in plasma significantly compared to the corresponding unformulated compound (45). Curcumin Phytosome™ is commercially available under the name of Meriva™ curcumin and has recently been shown to be effective in a variety of pathologies in several pilot studies (46–56).

Here, we show for the first time that chronic IL6 expression in the retina of the GFAP-IL6 mice was associated with a significant increase in microglial number and reactive morphology, as well as neuronal loss. Furthermore, we show that GFAP-IL6 mice fed with 4.4 mg/g curcumin Phytosome^TM^, showed significantly fewer microglia and reactive phenotypes, and reduced neuronal loss.

## 2 Materials and methods

### 2.1 Animals

Animals were caged in groups of 2-5 mice per cage, in the animal facility of the School of Medicine, Western Sydney University under a 12 hours light (300 lx): 12 hours dark cycle at 23°C, 60% humidity. Food and water were provided *ad libitum*. The experimental procedures were approved by Western Sydney University Animal Care and Ethics Committee (approval ID: A11393) and carried out in accordance with the rules established by the National Health and Medical Research Council of Australia.

Normal food pellets were supplied to wild type (WT, C57BL/6) and heterozygous GFAP-IL6 mice of both genders weighing 20-30 grams, using both sexes. Three cohorts of GFAP-IL6 mice were fed with pellets containing 4.4, 2.2, or 1.1 mg/g of curcumin Phytosome^TM^ (Meriva^™^, Indena SpA, Italy) incorporated into their feed for a month (n=7/cohort) as described in (57). Curcumin Phytosome™ was supplied by Indena S.p.A, Italy, and incorporated into the mouse chow (Specialty Feeds, Perth, Australia). Curcumin was mixed with powdered mouse food, and around 15% water was added before introduced the mixed diet to the pellet machine. In the pelleting machine, the temperature of the product would not exceed 43C. After pelleting the moisture content had to be reduced to stabilize the diet against microbial storage damage. This was done by loading the pellets into a shallow tray in an air dryer set at 65C for 3 h. Over the drying cycle the temperature of the pellets was increased slowly from 25C to 45C. The mouse chow pellets were stored at room temperature in vacuum packed bags in the animal housing facility at the School of Medicine, Western Sydney University. The cage food trays were monitored weekly to record food consumption as previously described (57).

### 2.2 Immunohistochemistry

Mice were anaesthetized with pentobarbitone and transcardially perfused with 4% paraformaldehyde in 0.1 M PBS. Eyes were removed and the retinae isolated for wholemount preparation, or retinal slices:

#### 2.2.1 Whole mounts

Retinae were isolated and 4 relaxing cuts were made circumferentially. Free-floating tissue was blocked with 5% (Jackson ImmunoResearch, #017000121) and 3% tris-Triton-X-100 (Sigma Aldrich, # 9036-19-5) solution for 2 hours. Tissue was then incubated at room temperature for 72 hours with the primary antibody in 3% tris-triton solution and 1% donkey serum (Jackson ImmunoResearch, #017000121). Tissue was rinsed in 0.1% tris-Triton solution for 15 minutes three consecutive times. Respective secondary antibodies in 3% tris-Triton solution and 2% donkey serum (Jackson ImmunoResearch, #017000121) were applied to this tissue and incubated for 3 hours. Tissue was subsequently rinsed in 0.1% tris-Triton solution for 15 minutes three consecutive times. Wholemount tissue was then mounted on slides and sealed with Vectashield^®^ (Vectorlabs, #H-1000).

#### 2.2.2 Slices

For retinal slices, cornea and lens were removed from the eyes and the remaining eye-cups cryoprotected in 30% sucrose overnight and then immersed in OCT medium and frozen on liquid nitrogen. 15 μm sections were cut from the central retina 1 mm dorsal and ventral of the optic nerve and adhered to gel-coated microscope slides. The tissue was blocked with 5% donkey serum (Jackson ImmunoResearch, #017000121) and 0.3% tris-Triton-X-100 solution for 2 hours and then incubated at room temperature for ~18 hours with the primary antibody in 0.3% tris-Triton solution and 1% donkey serum (Jackson ImmunoResearch, #017000121). Tissue was rinsed in 0.1% tris-Triton solution for 5 minutes three consecutive times. Respective secondary antibodies in 0.3% Triton (Sigma Aldrich, # 9036-19-5) solution and 2% donkey serum (Jackson ImmunoResearch, #017000121) were applied to this tissue and incubated for 1.5 hours. Slides were subsequently rinsed in 0.1% tris-Triton solution for 5 minutes three consecutive times and then sealed with Vectashield^®^ (Vectorlabs, #H-1000).

#### 2.2.3 Antibodies

Primary antibody dilutions: rabbit anti-IBA 1:1000, Wako, #019-1974; rabbit anti-M-opsin, 1:500, Millipore #AB5407; mouse anti-rhodopsin 1:500, Millipore, #MAB5356; GFAP; 1:500, Millipore #MAB360. Secondary antibody (and fluorescent tag) dilutions: donkey anti-mouse TRITC (1:100; Jackson, #712025150), donkey anti-rabbit FITC (1:100; Jackson, #711095152), donkey anti-rabbit TRITC (1:100; Jackson, #711025152), FITC conjugated peanut agglutinin (PNA, 1:100; Vector Lab, #FL1071).

### 2.3 Counting and reconstruction

Mounted wholemount retinae were examined using a Zeiss AxioImager M2 microscope equipped with MBF Biosciences StereoInvestigator. The number of Iba1^+^ cells counted through the entire thickness of the tissue was divided by the area of the retina (calculated by contouring the whole retina at 10x magnification) to obtain a density value (Iba1^+^ cells per mm^2^). For reconstruction, Z-stack images of 1 µm thickness/stack were taken in a Zeiss LSM 5 confocal microscope. Images were examined using Neurolucida 360 (MBF Biosciences, VT, USA) software package and the processes were reconstructed using directional kernels mode was used for 3D reconstruction. Nuclei were manually reconstructed in 2D.

For each stained retinal slide, 5 images were taken under the confocal microscope from central to nasal/temporal. Densities of M opsin and PNA expression were counted in each and averaged. For measurements of thickness of the rhodopsin and nuclear layers, 3 points were taken on each image and all data points were averaged.

### 2.4 Analysis

All data is presented as mean ± SEM using GraphPad Prism 9. One-way analysis of variance (ANOVA) with Tukey’s post-test was used to compare untreated GFAP-IL6 with the treatment doses.

## 3 Results

Retinae of wild type (WT), GFAP-IL6, and GFAP-IL6 curcumin-fed animals were analyzed in both retinal slices and wholemount to determine the numbers of Iba1^+^ microglia, retinal architecture, cell density and layer thickness, and the potential of curcumin Phytosome^TM^ to protect against these inflammatory and degenerative changes.

### 3.1 Retinal microglial density is upregulated by chronic inflammation and can be reversed by curcumin Phytosome^TM^ supplementation

**Figure 1:**
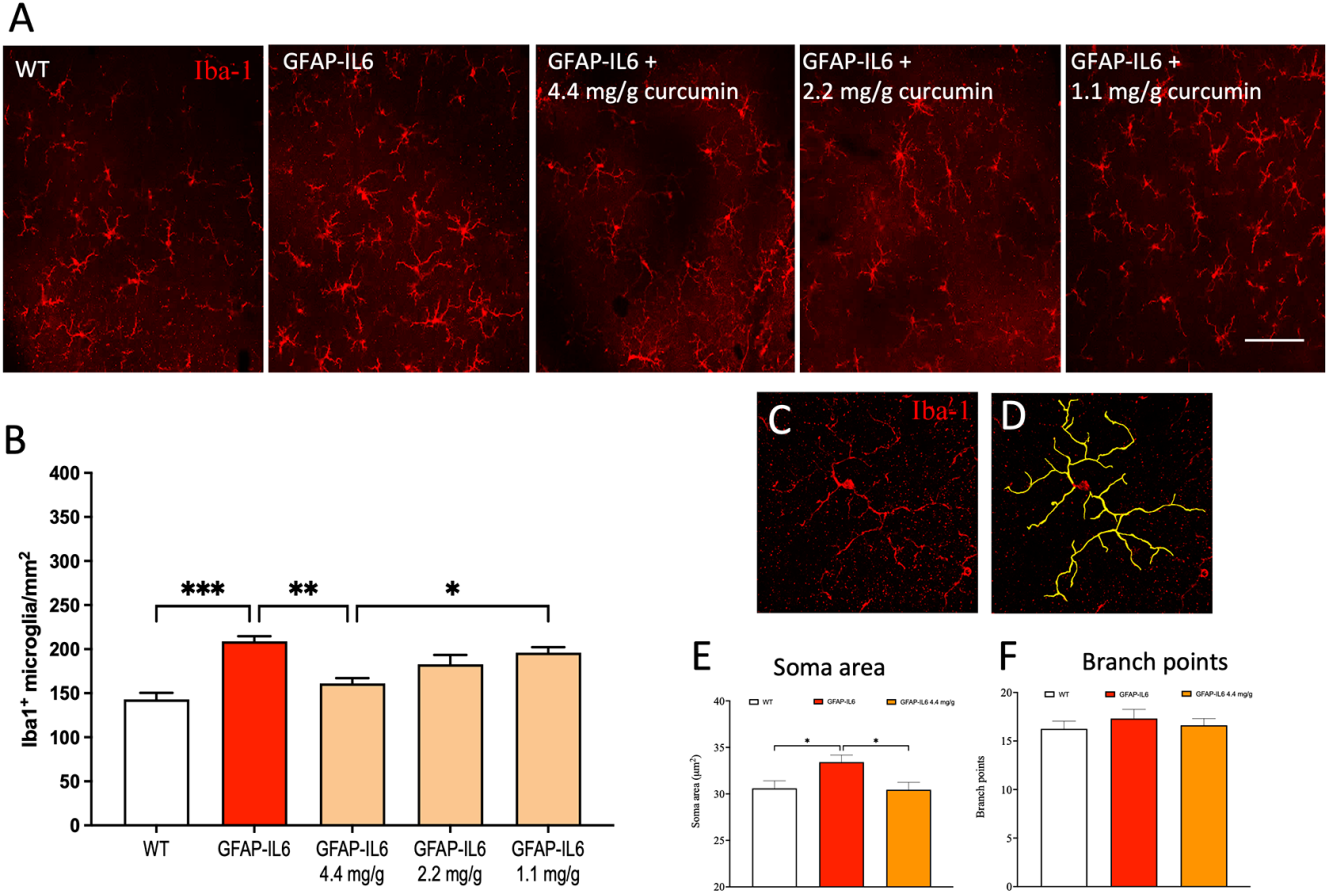
Curcumin Phytosome^TM^ reduces microglia numbers and the soma size, and the cell are area in the retina of the GFAP-IL6 mice but does not modify the location of the microglia. **(A)** Representative photographs of the retina labelled with Iba-1 antibody to visualize microglia (red) microglial cells at 20x magnification in WT, GFAP-IL6 and GFAP-IL6 (4.4, 2.2, or 1.1 mg/g curcumin treated animals. *Scale bar:50 µm.* **(B)** The retina of the GFAP-IL6 mice was found to be significantly amplified (***p < 0.001, n = 7) compared to their WT counterparts. A dose-response (4.4, 2.2, or 1.1 mg/g) of curcumin Phytosome^TM^ correlates with a dose-dependent reduction of absolute microglial numbers of the retina in GFAP-IL6 mice, but only the highest dose significantly reduces microglial density back to WT levels (***p < 0.001, *n* = 7). **(C, D)** An example of 3D morphological analysis was completed on confocal Z-stack image from an Iba1^+^ (red) microglial cell (*n* = 10 cells per animal = min of 65 cells per cohort) at 40x magnification resulting in 3D reconstructions (yellow). **(E)** GFAP-IL6 mice have an increased soma perimeter compared to WT, which is restored back to WT levels with 4.4 mg/g dose of curcumin however **(F)** the number of branch points were not affected by either genotype or treatment. *: p < 0.05; **: p < 0.01.

To investigate the differences in retinal microglial reactivity between the WT and GFAP-IL6 animals and the effect of curcumin Phytosome^TM^, analysis of Iba-1^+^ microglial density and morphology was performed in the retina in WT and GFAP-IL6, and GFAP-IL6 mice fed with different doses of curcumin Phytosome^TM^ for 1 month from 3-4 months of age. Firstly, we determined microglial density in the retinae. We found a significant difference between our groups [F (4,41) = 10.1, *p* < 0.0001]. Using multiple comparisons, we found that GFAP-IL6 mice exhibited significantly higher Iba-1^+^ microglia cell density (209 ± 11.2 cells/mm^2^) as compared to wild-type animals (143 ± 7.5 cells/mm^2^; mean ± SEM) (**Figures 1A, B** **)**.

Importantly, microglial density was significantly reduced back to WT levels (161 ± 6.08 cells/mm^2^) in mice fed with curcumin Phytosome^TM^ at a dose of 4.4 mg/g (**p < 0.002; (**Figures 1A, B** **)** suggesting a significant anti-inflammatory effect of this compound. The location of microglia within retinal sections was found to be similar for all experimental groups with cells located predominately in the inner and outer plexiform layers.

### 3.2 Microglial soma area is altered in response to both chronic inflammation and curcumin supplementation in the retina

To investigate individual morphological differences in retinal microglia, we used Z-stack images of single microglia and reconstructed them individually (**Figures 1C, D**). The classic morphological characterization of microglia showed typical signs of a reactive state in GFAP-IL6 mice. We measured the soma area and the number of branch points from our cohorts and analyzed the data. The soma area of the microglial cells in the retinae of the groups showed significant differences [F (2,239) = 4.89, *p* < 0.002]. Using multiple comparisons, we found that the GFAP-IL6 mice displayed significantly (*p* < 0.004) larger soma areas (33.4 ± 0.75 µm^2^) compared to their WT counterparts (30.6 ± 0.8µm^2^). This reactive morphology was reduced back to normal levels after curcumin Phytosome^TM^ treatment (30.4 ± 0.8 µm^2^) (**Figure 1E**), suggesting that curcumin not only reduces retinal microglial density but returns them to their non-reactive state. While we also analyzed the number of branching points of the dendrites, we found no significant differences between the groups (**Figure 1F**).

### 3.3 Cone density is reduced in GFAP-IL6 retinae which can be prevented by curcumin supplementation

**Figure 2:**
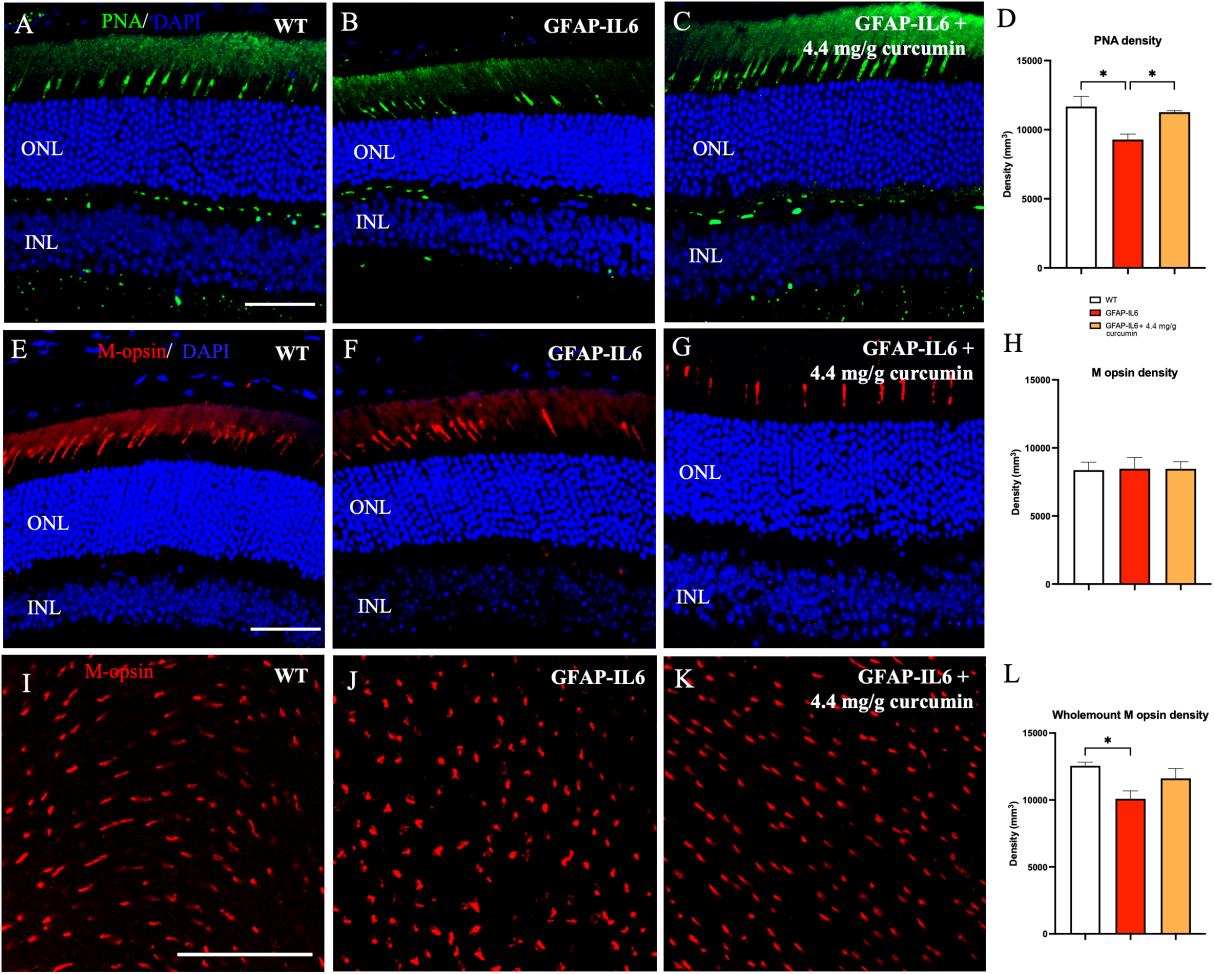
Cone density is reduced in the retina of GFAP-IL6 mice which can be prevented by curcumin treatment. **(A–D)** Significant decrease in PNA density sections between WT and GFAP-IL6 mice. This reduction was prevented in curcumin treated retinae. (*p < 0.05, *n* = 7) **(E–H)** No significant difference in M-opsin cone density was observed across the retinal slices between WT, GFAP-IL6 and curcumin treated retinae. **(I–L)** However, when we examined the whole mount sections, we found significant difference in M-opsin cone density between WT and GFAP-IL6, and curcumin treatment had no significant impact on the retinae. (*p < 0.05, *n* = 7). *Scale bars:50 µm.*.

Cones were stained with the lectin peanut agglutinin (PNA), which stains both cone types (S- and M-cones), to assess changes in density and morphology. We found significant differences in cone density between cohorts [F (2,10) = 7.8, *p* < 0.009]. Multiple comparisons between groups revealed that cone density, illustrated in **Figures 2A–C**, showed a significant decrease in the GFAP-IL6 retina when compared to WT retina (11,672 ± 731 vs 9,289.80 ± 390, adjusted *p* < 0.01). Curcumin treatment prevented this reduction in cone density when compared to unfed GFAP-IL6 retina (11,270 ± 108 adjusted *p* = 0.023) suggesting a significant neuroprotective effect of curcumin Phytosome^TM^ (**Figure 2D**).

To identify the specific type of cone affected by inflammation, retinae were stained with the M-opsin antibody. Both sections and whole mount staining was completed in WT, GFAP-IL6, and GFAP-IL6 curcumin fed mice. On examining retinal sections, (**Figures 2E–H**) we found no significant change in M-cone density between WT, GFAP-IL6, and curcumin treated [F (2, 10) = 0.0076, *p* > 0.99] (sections: 8370.10 ± 576.80 vs 8473.35 ± 828.76 vs 8465.88 ± 545.28, p=0.99). However, on examination of retinal wholemounts, we found significant differences in M-cone density between our cohorts [F (2,23) = 4.646, *p* = 0.023], with the multiple comparisons showing significant differences between the genotypes (WT 12,553 ± 278 vs GFAP-IL6 10,096 ± 581; *p* = 0.019). However, the curcumin treatment did not significantly reverse these differences (*p* = 0.17) (**Figures 2I–L**). While this data shows that M-cones are affected by an increase in inflammation, it does not completely explain the reduction in total cone density observed in slices. Therefore, it may be that the effect of inflammation affects S-cones more than M-cones. Future work will specifically quantify the number of cones expressing S-opsin.

### 3.4 Chronic inflammation causes a reduction in nuclear layer thickness that can be prevented by curcumin supplementation

**Figure 3:**
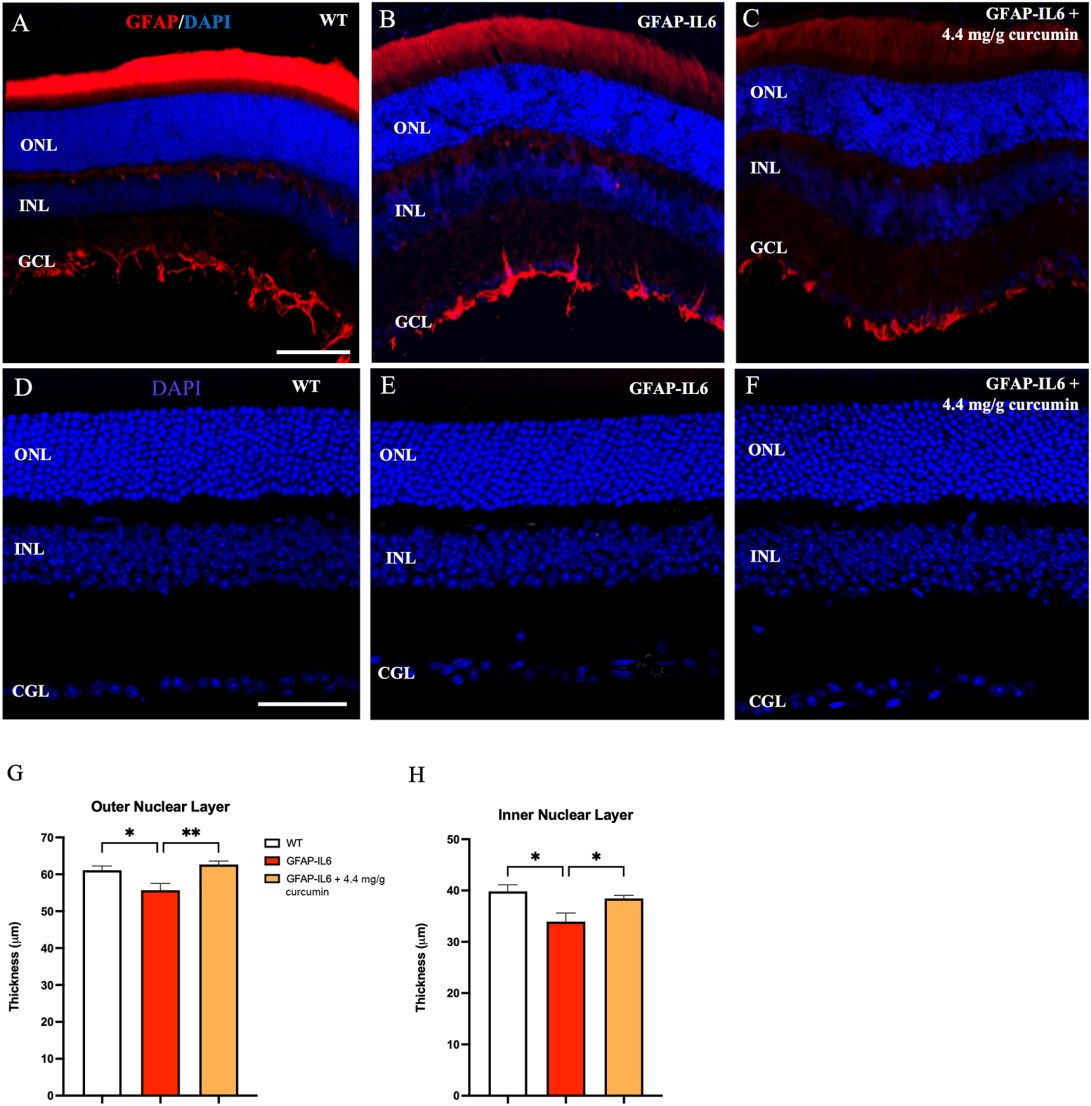
Nuclear layer thickness is reduced in the retina of GFAP-IL6 mice which can be prevented by curcumin treatment. **(A–C)** No GFAP expression in Müller cells was detected in wild-type and treated/untreated GFAP-IL6 animals. GFAP expression was confined to the inner limiting membrane. Inflammation causes a reduction in nuclear layer thickness which is prevented by curcumin supplementation. **(D–F)** Representative images of WT, GFAP-IL6 and GFAP-IL6 fed with 4.1 mg/g curcumin Phytosome^TM^, showing decreased outer and inner nuclear layers between WT and GFAP-IL6 animals, that was recovered after curcumin treatment. We found significant reduction in ONL **(G)** (*p < 0.05, *n* = 7; **p < 0.01, *n* = 7) and INL **(H)** (*p < 0.05, *n* = 7) thickness sections between WT and GFAP-IL6 sections. This nuclear layer thickness reduction was prevented by curcumin Phytosome^TM^ supplementation. **(A–C)** *Scale bar:100 µm.* **(D–F)** *Scale bar:50 µm*.

Given that these effects of chronic IL6 release originate from astroglia, presumably within the retina, we turned our attention to characterizing astroglial morphology, density, and location within the retina in wild-type, GFAP-IL6, and GFAP-IL6 curcumin fed animals. However, GFAP expression is only observed at the vitreal border of the inner limiting membrane (ILM) likely depicting extra-retinal astrocytes. No obvious Müller cell staining was apparent in sections for any experimental group (**Figures 3A–C**).

If cone numbers are reduced in GFAP-IL6 mice, destruction of photoreceptors should subsequently lead to a similar decrease in the cell bodies of those cells. These cell bodies are located in the outer (ONL). To investigate nuclear layer changes in thickness, nuclei were stained with DAPI and the thickness of both the ONL and INL were assessed (**Figures 3D–F**). We found a small but significant decrease in both the ONL [F (2,10) = 7.73, *p* < 0.009] and INL [F (2,10) = 6.52, *p* < 0.01] thickness when we comparing our cohorts. Using multiple comparisons, we found that the GFAP-IL6 mice had significantly thinner ONL (55.70 ± 1.83, *p* = 0.04) and INL (33.9 ± 1.67, *p* = 0.016) compared to their WT counterparts (61.12 ± 1.15 and 39.8 ± 1.27, respectively) (**Figures 3G, H** **)**. When retinal section thickness from curcumin-treated GFAF-IL6 mice (62.7± 0.9) was compared to the unfed GFAP-IL6 group (38.4 ± 0.61), the reduction in the ONL and INL thickness was eliminated (*p* = 0.008 and *p* = 0.049, respectively) indicating significant neuroprotection by curcumin against inflammation.

### 3.5 Rod outer segment length is not impacted by increased IL-6 expression

**Figure 4:**
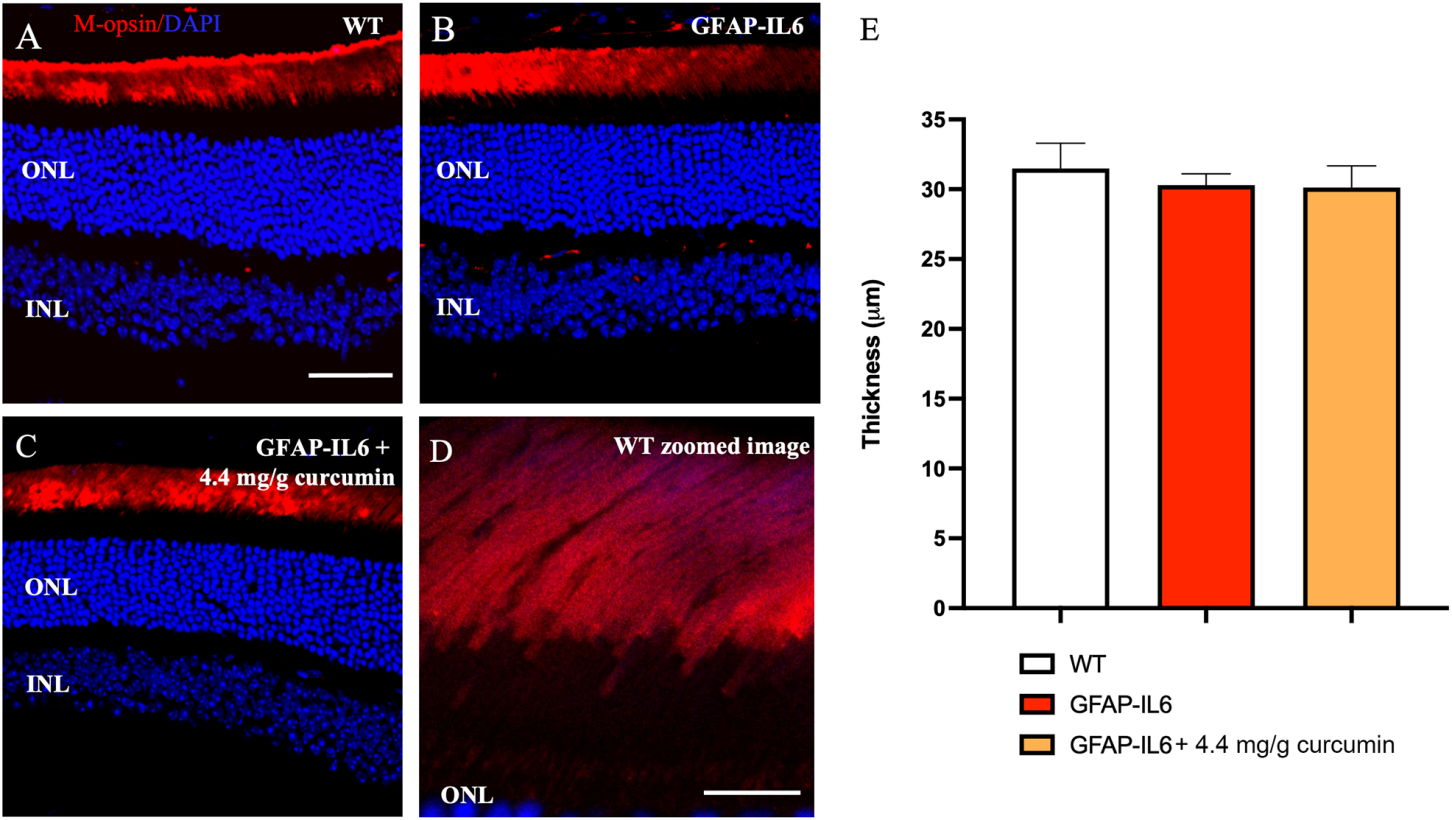
Rod outer segment thickness is not impacted by inflammation. No significant quantitative difference in rhodopsin layer thickness was seen between sections. **A–D)** Representative images of the WT, GFAP-IL6 and GFAP-IL6 4.4 mg/g treated group. **(E)** Column statistics of the rod outer segment thickness showed no significant differences (n = 7) between groups. **(A–C)** *Scale bar:50 µm.* **(D)** *Scale bar:10 µm*.

To determine the impact of inflammation on rod photoreceptor outer segment length, the retinae were stained for rod opsin (**Figures 4A–D**). We found no significant differences when comparing the length of rod outer segments in WT, GFAP-IL6 and curcumin fed mice (31.49 ± 1.80 vs 30.31 ± 0.80 vs 30.12 ± 1.56, *p* = 0.79) (**Figure 4E**).

## 4 Discussion

As Iba-1^+^ microglia numbers are upregulated in the cerebellum and hippocampus of the GFAP-IL6 mice (30, 31, 57), we investigated if these inflammatory changes extend to the retina. For the first time, we have characterized microglial activation in GFAP-IL6 mice compared to age-matched wild-type animals and the effect of these neuroinflammatory processes on retinal architecture. We showed that the retina displays distinct hallmarks of neuroinflammation with an increased density of Iba-1+ microglia and reactive-like microglial morphology in the GFAP-IL6 mice. Further, we showed that cone density and retinal thickness are significantly reduced by chronic neuroinflammation. We found that highly bioavailable curcumin Phytosome^TM^ reversed these inflammatory changes in glial density and morphology and prevented the disruptions to the retinal architecture. In the retina, Iba-1 labels both microglia and choroidal macrophages and recruited monocytes (collectively called mononuclear phagocytes), therefore it is not possible to differentiate between these two populations with our methodologies (58) and as such our labeling may include a diverse cell population. Future studies will differentiate between these two populations and their respective impact on retinal architecture/function.

Regarding the bioavailability of this specific curcumin Phytosome^TM^ preparation, previous studies have shown an increased absorption in a range between 3 and 29-fold when curcumin was encapsulated in phospholipid complexes (45, 59), and therefore it is likely our results are enhanced in comparison to unencapsulated curcumin. Indeed, the stability in plasma of encapsulated curcumin up to 12 hours after ingestion is 6-fold higher than normal curcumin (60), and age appears to play an important role in the stability of curcumin in the plasma, lasting longer in aged mice (61). For instance, a study was performed with the same curcumin Phytosome^TM^ preparation (2 tablets a day) as an adjuvant to treat uveitis, and patients showed fewer relapses during the follow up year of treatment (62). Whether the effect of curcumin on the retina is direct, or extra-retinal (e.g. via the gut-brain axis by regulation of microbiota (63)), remains to be found. We found here that highly bioavailable curcumin Phytosome^TM^ had a substantial protective effect on the retinae of GFAP-IL6 mice, restoring microglial cell density to wild type levels and preventing neuronal cell loss.

While this is the first study to date on the retina of the GFAP-IL6 mice, other rodent models of retinal inflammation have also shown a similar phenotype. Models that mimic autoimmune uveoretinits by immunization of the animals with the retinal antigen, interphotoreceptor retinoid-binding protein (IRBP), show increased expression of inflammatory cytokines (IFN-γ, IL-6, and IL-17) in the retina, resulting in increased numbers of GFAP positive cells (astrocytes) and Iba-1 positive cells with activated morphology (64, 65). Further, a mouse model lacking the immune suppressing cytokine interleukin-33 (IL-33) was shown to display enhanced retinal degeneration and Müller cells gliosis following retinal detachment. The authors suggest that the exacerbation of this phenotype was a consequence of sustained subretinal inflammation from inflitrating macrophages (66). These data suggest that modulation of retinal cytokines plays a critical role in maintaining retinal health. The extent of the retinal phenotype we see in the GFAP-IL6 animals, while substantial enough to display significant retinal changes, is milder than these other mouse models that display significant retinal degeneration. The phenotype of GFAP-IL6 animals likely depicts the early stages of retinal inflammation and retinal destruction meaning it is an important model with which to study early interventions that may prevent the irreversible damage caused by chronic inflammation.

While retinal inflammation is known to exacerbate retinal disorders, few studies have examined the effect of inflammation alone on neuronal architecture/cell loss. Okomoto et al. employed an endotoxin-induced mouse model of neuroinflammation (lipopolysaccharide injection (LPS)) and found a shortening of the rod outer segment length that was also associated with a reduction in both the a- and b-wave of the electroretinogram (ERG) (67). While we do not see changes in rod outer segment length in the GFAP-IL6 mice, it is likely that acute LPS-induced inflammation is a more potent inflammatory model. The authors did not, however, specifically assess cone numbers and so it is possible that cone loss may precede rod loss in response to retinal inflammation. The reduction in cone numbers we see in our study may reflect an early stage of photoreceptor loss. Mouse cone photoreceptors have three different expression patterns, 1) S-opsin only (genuine S-cones), 2) M-opsin only (genuine – M-cones), and 3) cones that express both opsins (dual cones) (68, 69). The majority of mouse cones are dual cones but genuine S-cones can be found in ventral retina and genuine M-cones in the dorsal retina (70). The lectin peanut agglutin labels all cones (71)and we find cone total cone numbers reduced in the GFAP-IL6 mice when retinal slices are analyzed. However, when cones expressing M-opsin are quantified in slices we do not see a significant difference in cone numbers. Wholemout quantification of M-opsin reveals a significant loss of M-cones that is not fully prevented by curcumin supplementation unlike PNA density that can be completely prevented by curcumin supplementation. These data, and the disparity in slice quantification, suggests genuine S-cones may be more affected by inflammation in this animal model, and curcumin supplementation may affect each cone class differently. All slices analyzed contained both dorsal and ventral retinae, and PNA and M-opsin were co-stained and quantified in the same slices, so it is unlikely the quantification was biased. Future studies will examine this hypothesis further by analyzing genuine S- and M-cone numbers and localization (dorsal vs ventral) in GFAP-IL6 retina. Indeed, S-cone loss specifically appears to be a feature associated with early diabetic retinopathy where patients acquire significant tritanomaly (72, 73). Curcumin therapy has certainly been explored in patients with diabetic retinopathy and appears to play a preventative role (43). Our results suggest that curcumin treatment directly prevents cone loss in response to inflammation, and this may be beneficial to patients in the early stages of diabetes to prevent or prolong the time until diabetic retinopathy onset.

In the normal retina, GFAP is expressed in astrocytes and the end-feet of Müller cells. When Müller glia are “activated” GFAP has been shown to be marker of gliosis (74, 75). However, we do not see any evidence of GFAP upregulation in the GFAP-IL6 mice. The GFAP promoter has been shown to reliably drive expression of GFP in Müller cells (76) so it is likely that IL6 is released from Müller cells in the GFAP-IL6 transgenic animals as well as from extraretinal astrocytes that express GFAP (**Figure 3**). The upregulation of GFAP in Müller cells in the retina has only previously been shown in cases of substantial retina degeneration (77) and thus is likely a marker of potent inflammation/degeneration. The fact that we do not see GFAP expression in Müller cells agrees with our hypothesis that the GFAP-IL6 animal represents a mild, or early-onset case of retinal inflammation. Future work will examine if retinal ganglion cells are impacted more than photoreceptors given their close proximity to the GFAP-positive astrocytes.

Curcumin has many features that make it a strong therapeutic agent in many parts of the eye. Various reviews have summarized the anti-inflammatory potentials of curcumin against inflammation related pathologies, such as dry eye syndrome where curcumin reduced inflammation (43, 78). In animal models of cataracts, curcumin was found to reduce the free radical damage and calcium influx that is responsible for the proteolysis causing clouding of the lens (79, 80). In diabetic retinopathy, curcumin was found to improve retinal microcirculation by inducing nitric oxide production. For age-related macular degeneration (AMD), curcumin may provide therapy through beneficial effects on microglial cells that are responsible for drusen formation (43, 78). Other have also shown that curcumin was able to downregulate retinal inflammation and prevent cell death: Zhang et al. described the reduction of the expression of interleukin-17 (IL-17) and IL-23 in Sprague-Dawley rat retina after retinal ischemia-reperfusion injury due to the administration of curcumin in a dose dependent manner (81, 82). In addition, in a rat model of anterior uveitis, induced by LPS, the application of curcumin/calix[4]arene decreased ocular inflammation and reduced inflammatory proteins to a greater extent than free curcumin (83). To treat glaucoma, curcumin-loaded nanoparticles in combination with latanoprost, a drug to increase uveoscleral outflow, were delivered using a thermosensitive chitosan-gelatin-based hydrogel, and showed reduced levels of inflammation-related genes (TNF, IL-1α, IL-6 and MMP-13), apoptosis, and ROS expression (84). Curcumin has been shown to possess potent anti-inflammatory properties by inhibiting various pro-inflammatory pathways. It can suppress the activation of pro-inflammatory transcription factor such as nuclear factor-kappa B (NF-kB), cytokines (e.g., interleukin-1β, tumor necrosis factor-alpha), and enzymes (e.g., cyclooxygenase-2, inducible nitric oxide synthase). Curcumin has also been found to inhibit pro-inflammatory signaling pathways related to neuroinflammation, such as Janus kinase/signal transducer and activator of transcription (JAK/STAT), mitogen-activated protein kinases (MAPKs), and phosphoinositide 3-kinase/protein kinase B (PI3K/Akt) (85, 86). By reducing proinflammatory signaling pathways, curcumin may help mitigate neuroinflammation associated with neurodegeneration, and therefore protect cone cells from damage and neuronal death. Furthermore, curcumin exhibits antioxidant properties, enabling it to reduce oxidative stress. Reactive oxygen species play a role in neuroinflammation and in neurodegenerative processes (87). By neutralizing reactive oxygen species (ROS) and enhancing antioxidant defenses, curcumin may protect neurons from oxidative damage. Finally, curcumin can modulate the Nrf2-ARE pathway, leading to increased expression of antioxidant enzymes and an increase in glutathione (88, 89).

Altogether, this study provides new insights into the potential role of chronic neuroinflammation in retinal damage and vision loss. We highlight the potential of encapsulated curcumin to ameliorate the symptoms caused by overactivation of microglia derived from retinal chronic inflammation. These data suggest that early interventions, such as curcumin supplementation, before retinal degeneration occurs, may be vital to prevent neuronal loss and dysfunction.

## Data availability statement

The original contributions presented in the study are included in the article/supplementary materials. Further inquiries can be directed to the corresponding author.

## Ethics statement

The animal study was approved by Western Sydney University Animal Care and Ethics Committee. The study was conducted in accordance with the local legislation and institutional requirements.

## Author contributions

GM and MC devised the experiments, edited and corrected the analysis and the manuscript. VP-F and AT completed immunohistochemistry, generated and analyzed the data, drafted the manuscript cell reconstruction and data analysis. FU fed and perfused the mice. EG planned the cohorts, supervised the breeding and feeding of the cohorts, and tissue collection, edited and corrected analysis and finalized manuscript and the figures. All authors have reviewed the manuscript.

## References

[1] Madeira MH Boia R Santos PF Ambrósio AF Santiago AR. Contribution of microglia-mediated neuroinflammation to retinal degenerative diseases. Mediators Inflammation (2015) 2015. doi: 10.1155/2015/673090 PMC4385698 25873768

[2] Ramirez AI de Hoz R Salobrar-Garcia E Salazar JJ Rojas B Ajoy D. The role of microglia in retinal neurodegeneration: Alzheimer’s disease, Parkinson, and glaucoma. Front Aging Neurosci (2017). doi: 10.3389/fnagi.2017.00214 PMC5498525 28729832

[3] Silverman SM Wong WT. Microglia in the retina: roles in development, maturity, and disease. Annu Rev Vis Sci (2018). doi: 10.1146/annurev-vision-091517-034425 29852094

[4] Block ML Hong JS. Microglia and inflammation-mediated neurodegeneration: Multiple triggers with a common mechanism. Prog Neurobiology (2005) 76:77–98. doi: 10.1016/j.pneurobio.2005.06.004 16081203

[5] Perry VH Holmes C. Microglial priming in neurodegenerative disease. Nat Rev Neurol (2014). doi: 10.1038/nrneurol.2014.38 24638131

[6] Huang Z Zhou T Sun X Zheng Y Cheng B Li M. Necroptosis in microglia contributes to neuroinflammation and retinal degeneration through TLR4 activation. Cell Death Differ (2018). doi: 10.1038/cdd.2017.141 PMC5729519 28885615

[7] Noailles A Fernández-sánchez L Lax P Cuenca N. Microglia activation in a model of retinal degeneration and TUDCA neuroprotective effects. (2014) J Neuroinflamm. 11:1–15. doi: 10.1186/s12974-014-0186-3 PMC4221719 25359524

[8] Blank T Goldmann T Koch M Amann L Schön C Bonin M. Early microglia activation precedes photoreceptor degeneration in a mouse model of CNGB1-linked retinitis pigmentosa. Front Immunol (2018). doi: 10.3389/fimmu.2017.01930 PMC5760536 29354133

[9] London A Benhar I Schwartz M. The retina as a window to the brain - From eye research to CNS disorders. Nat Rev Neurol (2013) 9. doi: 10.1038/nrneurol.2012.227 23165340

[10] Rojas B Gallego BI Ramírez AI Salazar JJ de Hoz R Valiente-Soriano FJ. Microglia in mouse retina contralateral to experimental glaucoma exhibit multiple signs of activation in all retinal layers. J Neuroinflamm (2014). doi: 10.1186/1742-2094-11-133 PMC4128533 25064005

[11] Arroba AI Alcalde-Estevez E García-Ramírez M Cazzoni D de la Villa P Sánchez-Fernández EM. Modulation of microglia polarization dynamics during diabetic retinopathy in db/db mice. Biochim Biophys Acta - Mol Basis Dis (2016). doi: 10.1016/j.bbadis.2016.05.024 27267343

[12] Ahmed A Wang LL Abdelmaksoud S Aboelgheit A Saeed S Zhang CL. Minocycline modulates microglia polarization in ischemia-reperfusion model of retinal degeneration and induces neuroprotection. Sci Rep (2017). doi: 10.1038/s41598-017-14450-5 PMC5656679 29070819

[13] Zeng H Green W Tso M. Activation of microglia in human retinitis pigmentosa. Invest Ophthalmol Vis Sci (2005) 46(13):513. doi: 10.1016/s0014-4835(02)00332-9

[14] Grigsby JG Cardona SM Pouw CE Muniz A Mendiola AS Tsin ATC. The role of microglia in diabetic retinopathy. J Ophthalmol (2014). doi: 10.1155/2014/705783 PMC4166427 25258680

[15] Walker FR Beynon SB Jones KA Zhao Z Kongsui R Cairns M. Dynamic structural remodelling of microglia in health and disease: A review of the models, the signals and the mechanisms. Brain Behavior Immunity (2014) 37:1–14. doi: 10.1016/j.bbi.2013.12.010 24412599

[16] Davis BM Salinas-navarro M Cordeiro MF Moons L. Characterizing microglia activation: a spatial statistics approach to maximize information extraction. (2017) Sci Rep. 7:1–12. doi: 10.1111/j.1755-3768.2017.03623 28484229 PMC5431479

[17] Zhou T Huang Z Sun X Zhu X Zhou L Li M. Microglia polarization with M1/M2 phenotype changes in rd1 mouse model of retinal degeneration. (2017) Front Neuroanat. 11(September):1–11. doi: 10.3389/fnana.2017.00077 PMC5591873 28928639

[18] Zhou T Huang Z Zhu X Sun X Liu Y Cheng B. Alpha-1 antitrypsin attenuates M1 microglia-mediated neuroinflammation in retinal degeneration. Front Immunol (2018). doi: 10.3389/fimmu.2018.01202 PMC5988858 29899745

[19] Kalkman HO Feuerbach D. Antidepressant therapies inhibit inflammation and microglial M1-polarization. Pharmacol Ther (2016). doi: 10.1016/j.pharmthera.2016.04.001 27101921

[20] Park HJ Oh SH Kim HN Jung YJ Lee PH. Mesenchymal stem cells enhance α-synuclein clearance via M2 microglia polarization in experimental and human parkinsonian disorder. Acta Neuropathol (2016). doi: 10.1007/s00401-016-1605-6 27497943

[21] Zhou P Kannan R Spee C Sreekumar PG Dou G Hinton DR. Protection of retina by αB crystallin in sodium iodate induced retinal degeneration. PloS One (2014) 9(5). doi: 10.1371/journal.pone.0098275 PMC4038555 24874187

[22] Zhao L Zabel MK Wang X Ma W Shah P Fariss RN. Microglial phagocytosis of living photoreceptors contributes to inherited retinal degeneration. EMBO Mol Med (2015) 7(9). doi: 10.15252/emmm.201505298 PMC4568951 26139610

[23] Zabel MK Zhao L Zhang Y Gonzalez SR Ma W Wang X. Microglial phagocytosis and activation underlying photoreceptor degeneration is regulated by CX3CL1-CX3CR1 signaling in a mouse model of retinitis pigmentosa. Glia (2016) 64(9). doi: 10.1002/glia.23016 PMC4958518 27314452

[24] Lew DS Mazzoni F Finnemann SC. Microglia inhibition delays retinal degeneration due to merTK phagocytosis receptor deficiency. Front Immunol (2020) 11. doi: 10.3389/fimmu.2020.01463 PMC7381113 32765507

[25] Ozaki E Delaney C Campbell M Doyle SL. Minocycline suppresses disease-associated microglia (DAM) in a model of photoreceptor cell degeneration. Exp Eye Res (2022) 217. doi: 10.1016/j.exer.2022.108953 35090890

[26] Hu X Leak RK Shi Y Suenaga J Gao Y Zheng P. Microglial and macrophage polarization - New prospects for brain repair. Nat Rev Neurol (2015) 11. doi: 10.1038/nrneurol.2014.207 PMC4395497 25385337

[27] Campbell IL Abraham CR Masliah E Kemper P Inglis JD Oldstone MB. Neurologic disease induced in transgenic mice by cerebral overexpression of interleukin 6. Proc Natl Acad Sci U S A (1993) 90(21):10061–5. doi: 10.1073/pnas.90.21.10061 PMC47713 7694279

[28] Childs R Gamage R Münch G Gyengesi E. The effect of aging and chronic microglia activation on the morphology and numbers of the cerebellar Purkinje cells. Neurosci Lett (2021) 751. doi: 10.1016/j.neulet.2021.135807 33705934

[29] Chesworth R Gamage R Ullah F Sonego S Millington C Fernandez A. Spatial memory and microglia activation in a mouse model of chronic neuroinflammation and the anti-inflammatory effects of apigenin. Front Neurosci (2021) 15. doi: 10.3389/fnins.2021.699329 PMC8363202 34393713

[30] Ullah F Asgarov R Venigalla M Liang H Niedermayer G Münch G. Effects of a solid lipid curcumin particle formulation on chronic activation of microglia and astroglia in the GFAP-IL6 mouse model. Sci Rep (2020) 10(1). doi: 10.1038/s41598-020-58838-2 PMC7012877 32047191

[31] Gyengesi E Rangel A Ullah F Liang H Niedermayer G Asgarov R. Chronic microglial activation in the GFAP-IL6 mouse contributes to age-dependent cerebellar volume loss and impairment in motor function. Front Neurosci (2019) 13(APR). doi: 10.3389/fnins.2019.00303 PMC6456818 31001075

[32] Erta M Quintana A Hidalgo J. Interleukin-6, a major cytokine in the central nervous system. Int J Biol Sci (2012). doi: 10.7150/ijbs.4679 PMC3491449 23136554

[33] Campbell IL Hofer MJ Pagenstecher A. Transgenic models for cytokine-induced neurological disease. Biochim Biophys Acta - Mol Basis Disease (2010) 1802:903–17. doi: 10.1016/j.bbadis.2009.10.004 PMC2888861 19835956

[34] Ghasemi H. Roles of IL-6 in ocular inflammation: A review. Ocular Immunol Inflammation (2018). doi: 10.1080/09273948.2016.1277247 28146368

[35] Chen KH Wu CC Roy S Lee SM Liu JH. Increased interleukin-6 in aqueous humor of neovascular glaucoma. Investig Ophthalmol Vis Sci (1999) 40(11):2627–32. doi: 10.1016/j.preteyeres.2007.06.001.NEOVASCULAR 10509659

[36] Sims SM Holmgren L Cathcart HM Sappington RM. Spatial regulation of interleukin-6 signaling in response to neurodegenerative stressors in the retina. Am J Neurodegener Dis (2012) 1(2):168–79. PMC3560463 23024928

[37] Hae SL Ki KJ Jae YC Man HR Hong S Kwon M. Neuroprotective effect of curcumin is mainly mediated by blockade of microglial cell activation. Pharmazie (2007). doi: 10.1691/ph.2007.12.7563 18214347

[38] Karlstetter M Lippe E Walczak Y Moehle C Aslanidis A Mirza M. Curcumin is a potent modulator of microglial gene expression and migration. J Neuroinflamm (2011). doi: 10.1186/1742-2094-8-125 PMC3192695 21958395

[39] Yu Y Shen Q Lai Y Park SY Ou X Lin D. Anti-inflammatory effects of curcumin in microglial cells. Front Pharmacol (2018) 9(APR):1–10. doi: 10.3389/fphar.2018.00386 PMC5922181 29731715

[40] Kowluru RA Kanwar M. Effects of curcumin on retinal oxidative stress and inflammation in diabetes. Nutr Metab (2007). doi: 10.1186/1743-7075-4-8 PMC1868028 17437639

[41] Zhu W Wu Y Meng Y-F Wang J-Y Xu M Tao J-J. Effect of curcumin on aging retinal pigment epithelial cells. Drug Des Devel Ther (2015). doi: 10.2147/DDDT.S84979 PMC4590412 26445530

[42] Wang Y Yin Z Gao L Sun D Hu X Xue L. Curcumin delays retinal degeneration by regulating microglia activation in the retina of rd1 mice. Cell Physiol Biochem (2017). doi: 10.1159/000485085 29145208

[43] Peddada KV Brown A Verma V Nebbioso M. Therapeutic potential of curcumin in major retinal pathologies. Int Ophthalmol (2018). doi: 10.1007/s10792-018-0845-y 29404861

[44] Prasad S Tyagi AK Aggarwal BB. Recent developments in delivery, bioavailability, absorption and metabolism of curcumin: The golden pigment from golden spice. Cancer Res Treat (2014). doi: 10.4143/crt.2014.46.1.2 PMC3918523 24520218

[45] Cuomo J Appendino G Dern AS Schneider E McKinnon TP Brown MJ. Comparative absorption of a standardized curcuminoid mixture and its lecithin formulation. J Nat Prod (2011). doi: 10.1021/np1007262 21413691

[46] Appendino G Belcaro G Cornelli U Luzzi R Togni S Dugall M. Potential role of curcumin phytosome (Meriva) in controlling the evolution of diabetic microangiopathy. A pilot study. Panminerva Med (2011). 22108476

[47] Ledda A Belcaro G Dugall M Luzzi R Scoccianti M Togni S. Meriva??, a lecithinized curcumin delivery system, in the control of benign prostatic hyperplasia: A pilot, product evaluation registry study. Panminerva Med (2012). 23241931

[48] Hu S Belcaro G Dugall M Peterzan P Hosoi M Ledda A. Interaction study between antiplatelet agents, anticoagulants, thyroid replacement therapy and a bioavailable formulation of curcumin (Meriva^®^). Eur Rev Med Pharmacol Sci (2018) 22(15). doi: 10.26355/eurrev_201808_15647 30070343

[49] Steigerwalt R Nebbioso M Appendino G Belcaro G Ciammaichella G Cornelli U. Meriva^®^, a lecithinized curcumin delivery system, in diabetic microangiopathy and retinopathy. Panminerva Med (2012). 23241930

[50] Di Pierro F Rapacioli G Di Maio EA Appendino G Franceschi F Togni S. Comparative evaluation of the pain-relieving properties of a lecithinized formulation of curcumin (Meriva^®^), nimesulide, and acetaminophen. J Pain Res (2013). doi: 10.2147/JPR.S42184 PMC3596124 23526055

[51] Belcaro G Hosoi M Pellegrini L Appendino G Ippolito E Ricci A. A controlled study of a lecithinized delivery system of curcumin (meriva^®^) to alleviate the adverse effects of cancer treatment. Phyther Res (2014). doi: 10.1002/ptr.5014 23775598

[52] Antiga E Bonciolini V Volpi W Del Bianco E Caproni M. Oral curcumin (meriva) is effective as an adjuvant treatment and is able to reduce IL-22 serum levels in patients with psoriasis vulgaris. BioMed Res Int (2015). doi: 10.1155/2015/283634 PMC4450233 26090395

[53] Franceschi F Feregalli B Togni S Cornelli U Giacomelli L Eggenhoffner R. A novel phospholipid delivery system of curcumin (Meriva^®^) preserves muscular mass in healthy aging subjects. Eur Rev Med Pharmacol Sci (2016). 26957282

[54] Di Pierro F Zacconi P Bertuccioli A Togni S Eggenhoffner R Giacomelli L. A naturally-inspired, curcumin-based lecithin formulation (Meriva formulated as the finished product Algocur) alleviates the osteo-muscular pain conditions in rugby players. Eur Rev Med Pharmacol Sci (2017). doi: 10.4271/850177 29164565

[55] Pastorelli D Fabricio ASC Giovanis P D’Ippolito S Fiduccia P Soldà C. Phytosome complex of curcumin as complementary therapy of advanced pancreatic cancer improves safety and efficacy of gemcitabine: Results of a prospective phase II trial. Pharmacol Res (2018). doi: 10.1016/j.phrs.2018.03.013 29614381

[56] Mazzolani F Togni S Giacomelli L Eggenhoffner R Franceschi F. Oral administration of a curcumin-phospholipid formulation (Meriva(R)) for treatment of chronic diabetic macular edema: a pilot study. Eur Rev Med Pharmacol Sci (2018). doi: 10.26355/eurrev_201806_15189 29917217

[57] Ullah F Liang H Niedermayer G Münch G Gyengesi E. Evaluation of curcumin Phytosome^TM^ as an anti-inflammatory agent for chronic glial activation in the GFAP-IL6 mouse model. Front Neurosci (2020) 14. doi: 10.3389/fnins.2020.00170 PMC7081170 32226360

[58] Lückoff A Scholz R Sennlaub F Xu H Langmann T. Comprehensive analysis of mouse retinal mononuclear phagocytes. Nat Protoc (2017) 12(6). doi: 10.1038/nprot.2017.032 28471458

[59] Liu A Lou H Zhao L Fan P. Validated LC/MS/MS assay for curcumin and tetrahydrocurcumin in rat plasma and application to pharmacokinetic study of phospholipid complex of curcumin. J Pharm BioMed Anal (2006). doi: 10.1016/j.jpba.2005.09.032 16316738

[60] Jäger R Lowery RP Calvanese AV Joy JM Purpura M Wilson JM. Comparative absorption of curcumin formulations. Nutr J (2014). doi: 10.1186/1475-2891-13-11 PMC3918227 24461029

[61] Kocher A Hagl S Schiborr C Eckert GP Frank J. Concentrations of total curcuminoids in plasma, but not liver and kidney, are higher in 18-than in 3-months old mice. NFS J (2015). doi: 10.1016/j.nfs.2015.03.002

[62] Allegri P Mastromarino A Neri P. Management of chronic anterior uveitis relapses: Efficacy of oral phospholipidic curcumin treatment. Long-term follow-up. Clin Ophthalmol (2010). doi: 10.2147/OPTH.S13271 PMC2964958 21060672

[63] Shen L Liu L Ji H-F. Regulative effects of curcumin spice administration on gut microbiota and its pharmacological implications. Food Nutr Res (2017). doi: 10.1080/16546628.2017.1361780 PMC5553098 28814952

[64] Kudo A Keino H Sato Y Shoko S Nakayama M Sugita S. Genetic ablation of nrf2 exacerbates neuroinflammation in ocular autoimmunity. Int J Mol Sci (2022) 23(19). doi: 10.3390/ijms231911715 PMC9569802 36233013

[65] Rao NA Kimoto T Zamir E Giri R Wang R Ito S. Pathogenic role of retinal microglia in experimental uveoretinitis. Investig Ophthalmol Vis Sci (2003) 44(1). doi: 10.1167/iovs.02-0199 12506051

[66] Augustine J Pavlou S Ali I Harkin K Ozaki E Campbell M. IL-33 deficiency causes persistent inflammation and severe neurodegeneration in retinal detachment. J Neuroinflamm (2019) 16(1). doi: 10.1186/s12974-019-1625-y PMC6889479 31796062

[67] Okamoto T Ozawa Y Kamoshita M Osada H Toda E Kurihara T. The neuroprotective effect of rapamycin as a modulator of the mTOR-NF-KB axis during retinal inflammation. PloS One (2016) 11(1). doi: 10.1371/journal.pone.0146517 PMC4714903 26771918

[68] Haverkamp S Wässle H Duebel J Kuner T Augustine GJ Feng G. The primordial, blue-cone color system of the mouse retina. J Neurosci (2005) 25(22). doi: 10.1523/JNEUROSCI.1117-05.2005 PMC6725002 15930394

[69] Applebury ML Antoch MP Baxter LC Chun LLY Falk JD Farhangfar F. The murine cone photoreceptor: A single cone type expresses both S and M opsins with retinal spatial patterning. Neuron (2000) 27(3). doi: 10.1016/S0896-6273(00)00062-3 11055434

[70] Ortiń-Martínez A Nadal-Nicolás FM Jimeńez-López M Alburquerque-Béjar JJ Nieto-Loṕez L Garcia-Ayuso D. Number and distribution of mouse retinal cone photoreceptors: Differences between an albino (Swiss) and a pigmented (C57/BL6) strain. PloS One (2014) 9(7). doi: 10.1371/journal.pone.0102392 PMC4100816 25029531

[71] Williams GA Jacobs GH. Cone-based vision in the aging mouse. Vision Res (2007) 47(15). doi: 10.1016/j.visres.2007.03.023 PMC2049007 17509638

[72] Greenstein V Sarter B Hood D Noble K Carr R. Hue discrimination and S cone pathway sensitivity in early diabetic retinopathy. Investig Ophthalmol Vis Sci (1990) 31(6). doi: 10.2147/OPTH.S13271 2354907

[73] Cho NC Poulsen GL Ver Hoeve JN Nork TM. Selective loss of S-cones in diabetic retinopathy. Arch Ophthalmol (2000) 118(10). doi: 10.1001/archopht.118.10.1393 11030822

[74] Eisenfeld AJ Bunt-Milam AH Sarthy PV. Muller cell expression of glial fibrillary acidic protein after genetic and experimental photoreceptor degeneration in the rat retina. Investig Ophthalmol Vis Sci (1984) 25(11). doi: 10.1016/0304-3940(94)11239-F 6386743

[75] Bringmann A Pannicke T Grosche J Francke M Wiedemann P Skatchkov SN. Müller cells in the healthy and diseased retina. Prog Retinal Eye Res (2006) 25. doi: 10.1016/j.preteyeres.2006.05.003 16839797

[76] Kuzmanovic M Dudley VJ Sarthy VP. GFAP promoter drives Müller cell-specific expression in transgenic mice. Investig Ophthalmol Vis Sci (2003) 44(8). doi: 10.1167/iovs.02-1265 12882814

[77] Nork TM Ghobrial MW Peyman GA Tso MOM. Massive retinal gliosis: A reactive proliferation of müller cells. Arch Ophthalmol (1986) 104(9). doi: 10.1001/archopht.1986.01050210137041 3092790

[78] Pescosolido N Giannotti R Plateroti AM Pascarella A Nebbioso M. Curcumin: Therapeutical potential in ophthalmology. Planta Med (2014) 80. doi: 10.1055/s-0033-1351074 24323538

[79] Cao J Wang T Wang M. Investigation of the anti-cataractogenic mechanisms of curcumin through in *vivo* and in *vitro* studies. BMC Ophthalmol (2018) 18(1). doi: 10.1186/s12886-018-0711-8 PMC5816369 29454324

[80] Manikandan R Thiagarajan R Beulaja S Chindhu S Mariammal K Sudhandiran G. Anti-cataractogenic effect of curcumin and aminoguanidine against selenium-induced oxidative stress in the eye lens of Wistar rat pups: An in *vitro* study using isolated lens. Chem Biol Interact (2009) 181(2). doi: 10.1016/j.cbi.2009.05.011 19481068

[81] Zhang HJ Liang L. Effects of curcumin on structure and the expression of interleukin-23 and interleukin-17 in rat retinal ischemia-reperfusion injury. Int Eye Sci (2017) 17(8). doi: 10.3980/j.issn.1672-5123.2017.8.08

[82] Zhang HJ Xing YQ Jin W Li D Wu K Lu Y. Effects of curcumin on interleukin-23 and interleukin-17 expression in rat retina after retinal ischemia-reperfusion injury. Int J Clin Exp Pathol (2015) 8(8). PMC4583902 26464670

[83] Granata G Paterniti I Geraci C Cunsolo F Esposito E Cordaro M. Potential eye drop based on a calix[4]arene nanoassembly for curcumin delivery: enhanced drug solubility, stability, and anti-inflammatory effect. Mol Pharm (2017) 14(5). doi: 10.1021/acs.molpharmaceut.6b01066 28394618

[84] Cheng YH Ko YC Chang YF Huang SH Liu CJL. Thermosensitive chitosan-gelatin-based hydrogel containing curcumin-loaded nanoparticles and latanoprost as a dual-drug delivery system for glaucoma treatment. Exp Eye Res (2019) 179. doi: 10.1016/j.exer.2018.11.017 30471279

[85] Zhou X Venigalla M Raju R Münch G. Pharmacological considerations for treating neuroinflammation with curcumin in Alzheimer’s disease. J Neural Transm (2022) 129. doi: 10.1007/s00702-022-02480-x 35294663

[86] Venigalla M Gyengesi E Münch G. Curcumin and apigenin – Novel and promising therapeutics against chronic neuroinflammation in Alzheimer’s disease. Neural Regener Res (2015) 10(8). doi: 10.4103/1673-5374.162686 PMC4590215 26487830

[87] Calabrese V Bates TE Mancuso C Cornelius C Ventimiglia B Cambria MT. Curcumin and the cellular stress response in free radical-related diseases. Mol Nutr Food Res (2008) 52. doi: 10.1002/mnfr.200700316 18792015

[88] Yoo GY Kim E Kang H Kim J Yeo WS. Mass spectrometric investigation of concentration-dependent effect of curcumin and oxidative stress on intracellular glutathione levels. Anal Bioanal Chem (2020) 412(12). doi: 10.1007/s00216-020-02524-9 32112130

[89] Morris G Anderson G Dean O Berk M Galecki P Martin-Subero M. The glutathione system: A new drug target in neuroimmune disorders. Mol Neurobiol (2014) 50. doi: 10.1007/s12035-014-8705-x 24752591

